# Supplementary material for: Phylogenetic analysis of the distribution of deadly amatoxins among the little brown mushrooms of the genus Galerina
Source: PLoS One. 2021 Feb 10;16(2):e0246575. doi: 10.1371/journal.pone.0246575 (PMC7875387; doi:10.1371/journal.pone.0246575)
Supplement: S1 Fig — In this maximum likelihood tree numbers at nodes represent bootstrap support >50% from concatenated ITS, LSU and RPB2 data. Support values are omitted from some deeply nested clades due to graphic constraints. Light grey boxes show monophyletic, delimited Galerina species. Darker grey boxes show delimited but paraphyletic species. A species/clade name is given in each box. Sequence names from original identifications are followed by a voucher identifier. Where applicable, the number of collections from the same country with the same sequence is given in parentheses. +TOX in magenta, α-amanitin is present; -TOX in green, no amanitins were detected. Vertical lines designate subgenera as follows: Black, G. marginata s. l.; solid purple, Naucoriopsis; dashed purple, possible Naucoriopsis; green, Galerina; blue Tubariopsis; gold Mycenopsis; red Sideroides. Orange designates Gymnopilus spp. nested within Galerina. (DOCX) [file pone.0246575.s001.docx]

**S1 Fig. Phylogeny showing *Galerina* collections tested for amatoxins with species delimitations and country of provenance.** In this maximum likelihood tree numbers at nodes represent bootstrap support >50% from concatenated ITS, LSU and *RPB2* data. Support values are omitted from some deeply nested clades due to graphic constraints. Light grey boxes show monophyletic, delimited *Galerina* species. Darker grey boxes show delimited but paraphyletic species. A species/clade name is given in each box. Sequence names from original identifications are followed by a voucher identifier. Where applicable, the number of collections from the same country with the same sequence is given in parentheses. +TOX in magenta, α-amanitin is present; -TOX in green, no amanitins were detected. Vertical lines designate subgenera as follows: black, *G. marginata* s. l.; solid purple, *Naucoriopsis*; dashed purple, possible *Naucoriopsis*; green, *Galerina*; blue *Tubariopsis*; gold *Mycenopsis*; red Sideroides. Orange designates *Gymnopilus* spp. nested within *Galerina.*
