# Supplementary material for: Phylogenetic analysis of the distribution of deadly amatoxins among the little brown mushrooms of the genus Galerina
Source: PLoS One. 2021 Feb 10;16(2):e0246575. doi: 10.1371/journal.pone.0246575 (PMC7875387; doi:10.1371/journal.pone.0246575)
Supplement: S2 Fig — In this maximum likelihood tree of 368 sequences, thickened branches represent bootstrap support >50% from ITS data. Branch thickening is omitted from some deeply nested clades due to graphic constraints. Light grey boxes show monophyletic delimited Galerina species. Darker grey boxes show delimited but paraphyletic species. Sequences that are not boxed were less than 500 bp in length and not included in ABGD species delimitation. A species/clade name is given in each box. Sequence names from original identifications are followed by a voucher identifier and preceded by a number to help locate the same voucher in RPB2 and LSU gene trees. Vertical lines designate subgenera as follows: Black, G. marginata s. l.; solid purple, Naucoriopsis; dashed purple, possible Naucoriopsis; green, Galerina; blue Tubariopsis; gold Mycenopsis; red Sideroides. Orange designates Gymnopilus spp. nested within Galerina. (DOCX) [file pone.0246575.s002.docx]

**S2 Fig. Phylogeny of ITS sequences.** In this maximum likelihood tree of 368 sequences, thickened branches represent bootstrap support >50% from ITS data. Branch thickening is omitted from some deeply nested clades due to graphic constraints. Light grey boxes show monophyletic delimited *Galerina* species. Darker grey boxes show delimited but paraphyletic species. Sequences that are not boxed were less than 500 bp in length and not included in ABGD species delimitation. A species/clade name is given in each box. Sequence names from original identifications are followed by a voucher identifier and preceded by a number to help locate the same voucher in *RPB2* and LSU gene trees. Vertical lines designate subgenera as follows: black, *G. marginata* s. l.; solid purple, *Naucoriopsis*; dashed purple, possible *Naucoriopsis*; green, *Galerina*; blue *Tubariopsis*; gold *Mycenopsis*; red Sideroides. Orange designates *Gymnopilus* spp. nested within *Galerina.*
