# Supplementary material for: Phylogenetic analysis of the distribution of deadly amatoxins among the little brown mushrooms of the genus Galerina
Source: PLoS One. 2021 Feb 10;16(2):e0246575. doi: 10.1371/journal.pone.0246575 (PMC7875387; doi:10.1371/journal.pone.0246575)
Supplement: S3 Fig — In this maximum likelihood tree with 78 taxa, numbers at nodes represent bootstrap support >70% from RPB2 data. Support values are omitted from some deeply nested clades due to graphic constraints. Light grey boxes show monophyletic, delimited Galerina species. A species/clade name is given in each box. Sequence names from original identifications are followed by a voucher identifier and preceded by a number to help locate the same voucher in ITS and LSU gene trees. Vertical lines designate subgenera as follows: Solid purple, Naucoriopsis; dashed purple, possible Naucoriopsis; green, Galerina; blue Tubariopsis; gold Mycenopsis; red Sideroides. Orange designates Gymnopilus sapineus nested within Galerina. (DOCX) [file pone.0246575.s003.docx]

**S3 Fig. Phylogeny of *RPB2* sequences.** In this maximum likelihood tree with 78 taxa, numbers at nodes represent bootstrap support >70% from *RPB2* data. Support values are omitted from some deeply nested clades due to graphic constraints. Light grey boxes show monophyletic, delimited *Galerina* species. A species/clade name is given in each box. Sequence names from original identifications are followed by a voucher identifier and preceded by a number to help locate the same voucher in ITS and LSU gene trees. Vertical lines designate subgenera as follows: solid purple, *Naucoriopsis*; dashed purple, possible *Naucoriopsis*; green, *Galerina*; blue *Tubariopsis*; gold *Mycenopsis*; red *Sideroides*. Orange designates *Gymnopilus* *sapineus* nested within *Galerina.*
