# Supplementary material for: Phylogenetic analysis of the distribution of deadly amatoxins among the little brown mushrooms of the genus Galerina
Source: PLoS One. 2021 Feb 10;16(2):e0246575. doi: 10.1371/journal.pone.0246575 (PMC7875387; doi:10.1371/journal.pone.0246575)
Supplement: S4 Fig — In this maximum likelihood tree with 154 taxa, numbers at nodes represent bootstrap support >70% from LSU data. Support values are omitted from some deeply nested clades due to graphic constraints. Light grey boxes show monophyletic, delimited Galerina species. Darker grey boxes show delimited but paraphyletic species. A species/clade name is given in each box. Sequence names from original identifications are followed by a voucher identifier and preceded by a number to help locate the same voucher in ITS and LSU gene trees. Vertical lines designate subgenera as follows: Solid purple, Naucoriopsis; dashed purple, possible Naucoriopsis; green, Galerina; blue Tubariopsis; gold Mycenopsis; brown Sideroides. Orange designates Gymnopilus spp. nested within Mycenopsis. (DOCX) [file pone.0246575.s004.docx]

**S4 Fig. Phylogeny of LSU sequences.** In this maximum likelihood tree with 154 taxa, numbers at nodes represent bootstrap support >70% from LSU data. Support values are omitted from some deeply nested clades due to graphic constraints. Light grey boxes show monophyletic, delimited *Galerina* species. Darker grey boxes show delimited but paraphyletic species. A species/clade name is given in each box. Sequence names from original identifications are followed by a voucher identifier and preceded by a number to help locate the same voucher in ITS and LSU gene trees. Vertical lines designate subgenera as follows: solid purple, *Naucoriopsis*; dashed purple, possible *Naucoriopsis*; green, *Galerina*; blue *Tubariopsis*; gold *Mycenopsis*. Orange designates *Gymnopilus* spp. nested within *Galerina.*
