# Supplementary material for: Phylogenetic analysis of the distribution of deadly amatoxins among the little brown mushrooms of the genus Galerina
Source: PLoS One. 2021 Feb 10;16(2):e0246575. doi: 10.1371/journal.pone.0246575 (PMC7875387; doi:10.1371/journal.pone.0246575)
Supplement: S5 Fig — Color of names at the left designates delimited species: G. castaneipes, brown; G. venenata, blue; and G. marginata, red. Under a scenario of random interbreeding, frequent heterozygosity rather than private alleles would be expected. Instead, each of the three delimited species has unique nucleotide substitutions. (DOCX) [file pone.0246575.s005.docx]

**S5 Fig. Alignment of variable sites from the ITS region supports genetic separation of three species in *Galerina marginata* s.l.** Color of names at the left designates delimited species: *G. castaneipes*, brown; *G. venenata*, blue; and *G. marginata*, red. Under a scenario of random interbreeding, frequent heterozygosity rather than private alleles would be expected. Instead, each of the three delimited species has unique nucleotide substitutions.
