# Supplementary material for: Phylogenetic analysis of the distribution of deadly amatoxins among the little brown mushrooms of the genus Galerina
Source: PLoS One. 2021 Feb 10;16(2):e0246575. doi: 10.1371/journal.pone.0246575 (PMC7875387; doi:10.1371/journal.pone.0246575)
Supplement: S3 Table — (DOCX) [file pone.0246575.s008.docx]

S3 Table. Justification for the application of names to species.

| Subgenus or genus | ABGD group #; Species Name | Clade composition |
| --- | --- | --- |
| **Subgenus** *Naucoriopsis*  Kühner ex Gulden |  | *Naucoriopsis*: Mushrooms relatively large for *Galerina*, fleshy caps up to ~4 cm diam, margin initially inrolled, with a ring or fibrillose zone on stipe; often on wood; spores dextrinoid, plage distinct (Gulden et al., 2005). Pleurocystidia as well as cheilocystidia. Cystidia ventricose fuscoid or ventricose subcapitate (Gulden and Hallgrímsson, 2000)*.* Gulden & Hallgrímsson (2000) raised this widely recognized section to subgenus. Phylogenies suggest *Naucoriopsis* includes *G. jaapii*. and possibly, additional species designated with '*Naucoriopsis* (?)'. |
| *Naucoriopsis* | 51; *G.* aff. *marginata* | In *G. marginata* complex. Single sequence, nested in *G. marginata*. |
| *Naucoriopsis* | 36; *G. badipes* (Pers.) Kühner | Supported clade of 11 European and N. American sequences. Clade includes Smith types for *G. cedretorum* var. *microspora* and *G. cedretorum* var. *bispora*. *G. cedretorum* has been synonymized under '*G. badipes*. Two spores per basidium. Number of spores per basidium is inconsistent among sect. *Galerina* species (Gulden et al., 2005) and further investigation is needed to test how consistent the association is among *Naucoriopsis* species. |
| *Naucoriopsis* | 49; *G. castaneipes* A.H. Sm. & Singer | In *G. marginata* complex but never synonymized under *G. marginata*. Lacks a ring. Monophyletic without support; 21 sequences all west coast N. America; included is Smith *G. castaneipes* holotype sequence from Oregon. |
| *Naucoriopsis* | 18; *G. jaapii* A.H. Sm. & Singer | Supported clade, all European. |
| *Naucoriopsis* | 50; *G. marginata* (Batsch) Kühner | In *G. marginata* complex, Europe and N. America. Paraphyletic, 87% support for clade including ABGD group 50 and 51. This is one of several lineages that could be '*G. marginata*,' an old European species without a type. |
| *Naucoriopsis* | Number not assigned; short sequences, not grouped; *G. marginata* complex | Supported, in *G. marginata* complex, two sequences, Australia and N. America. Based on geography and small numbers, not likely '*G. marginata*,' an old European species without a type. |
| *Naucoriopsis* | 43; *G. makereriensis* Pegler | Single, divergent sequence. |
| *Naucoriopsis* | 44; *G. patagonica* Singer | In *G. marginata* complex. |
| *Naucoriopsis* | 45; *G. physospora* Singer  & *G. sulciceps* (Berk.) Boedijn | Supported clade of three. In *G. marginata* complex. *G. sulciceps* from China; *G. physospora* from Sao Tome, Africa. Need more samples to evaluate conspecificity. |
| *Naucoriopsis* | 1; *G. venenata* A.H. Sm. | In *G. marginata* complex. Not supported and paraphyletic. 38 sequences from Europe, North America. Named '*G. venenata'* because includes Smith type's sequence. Also includes type of *G. cinnamomea* var. *cinnamomea* – *G. venenata* older name (Smith, 1953). Correct name for clade is uncertain; this species (or species complex) is common and it could correspond to '*G. marginata*,' an old European species without a type. |
| *Naucoriopsis* (?) | 37; *G. chionophila* Senn-Irlet | Supported clade of two sequences with different identifications. Named *G. chionophila* because the second *G. harrisonii* collection O50711 is unrelated to clade 37 and is instead in *Mycenopsis* near the *Gymnopilus* sequences. |
| *Naucoriopsis* (?) | 3; *G. nana* sp. 1 | Supported clade of three slightly divergent N. American samples all identified as '*G. nana*.' |
| *Naucoriopsis* (?) | 20; *G. nana* sp. 2 | One slightly divergent, European sample, supported sister group to ABGD clade #3; clade #3 plus #20 monophyletic and all identified as '*G. nana*.' |
| *Naucoriopsis* (?) | 22; *G. pruinatipes* A.H. Sm. | Supported clade of 3 European and N. American samples, all identified as '*G. pruinatipes'*. Includes Smith holotype sequence (Gulden et al., 2005). |
| *Naucoriopsis* (?) | 23; *G. pseudocamerina* Singer | Supported clade of 4 sequences. Gulden's sequences identified as '*G. pseudocamerina*' (Gulden et al., 2005). Also in clade, *Galerina larigna* Singer 1945, which has priority over *G. pseudocamerina* Singer 1951. Smith examined the specimen LRHesler17642 (Smith and Singer, 1964) but unclear that ID of US specimen of this species described from Europe is correct. |
| *Naucoriopsis* (?) | 41; *G. salicicola* P.D. Orton  & *G. indica* K.P.D. Latha & Manim. | Supported clade, 2 samples. *G. indica* from India; *G. salicicola* from UK. Conspecific? Unclear without more data. |
| *Naucoriopsis* (?) | 2; *G. triscopa* (Fr.) Kühner | Supported clade of 4 samples. Name based on *G. triscopa* sequences (Gulden et al., 2005). |
| **Subgenus** *Galerina* |  | Subgenus *Galerina* is supported by concatenated data (90% bootstrap)*.* It includes *G. vittiformis* (Fr.) Singer, the generitype. Often on moss (Gulden et al., 2005). Cystidia as in *Naucoriopsis* but generally caulocystidia as well resulting in stipe that is pruinose over its length; also sometimes pileocystidia (Gulden and Hallgrímsson, 2000). Spores dextrinoid, plage distinct, as in *Naucoriopsis* (Gulden and Hallgrímsson, 2000). Gulden & Hallgrímsson (2000) included two sections in subgenus *Galerina*: *Galerina* and *Mycenopsis*. However, here, as in Gulden et al. (2005), subgenus *Galerina* appears as sister to *Naucoriopsis*, ruling out the inclusion of *Mycenopsis* in it. |
| *Galerina* | 57; *G. aff. vittiformis* sp. 4 | Supported clade of two specimens collected in Greenland (Gulden et al., 2005). In clade with '*G. vittiformis*' and all of the '*G. aff. vittiformis*' but also *G. minima and G. alpestris*. |
| *Galerina* | 56; *G. aff. vittiformis* sp. 2 | Supported clade, 3 of 4 samples identified as *G. atkinsoniana*  but not monophyletic with other *G. atkinsoniana* samples. Europe, Asia, N. America. |
| *Galerina* | 54; *G. aff. vittiformis* sp. 3 | Supported clade of 13 UBC *G. vittiformis* samples most from Ceska collections from Vancouver Island BC Canada. Nested in clade with '*G. vittiformis*' and all of the '*G. aff. vittiformis*' but also G. minima and G. alpestris. |
| *Galerina* | 59; *G. aff. vittiformis* sp. 5 | Supported clade of two *G. vittiformis* samples from Ceska collections from Vancouver Island BC Canada. Nested in clade with '*G. vittiformis*' and all of the '*G. aff. vittiformis*' but also *G. minima and G. alpestris.* |
| *Galerina* | 34; *G. alpestris* Singer | Supported clade of two samples from Italy. Nested in clade with '*G. vittiformis*' and all of the '*G. aff. vittiformis*' but sister to *G. minima*. |
| *Galerina* | 10; *G. atkinsoniana* A.H. Sm. | Supported sister clade to '*G. vittiformis*' and all of the '*G. aff. vittiformis*'. American and European specimens identified as *G. atkinsoniana* or *vittiformis*. Gulden et al. (Gulden et al., 2005): *atkinsoniana* is 2-4 spored with pileocystidia. No pileocystidia, is *vittiformis* or *minima*. Whitish veil remains, is *G. minima*. But Gulden et al. point out that these characters didn't work based on inconsistent specimen ids. |
| *Galerina* | 58; *G. minima* (Peck) A.H. Sm. & Singer | Supported clade of three Nordic samples, two identified as '*G. minima*'. Nested in clade with '*G. vittiformis*' and all of the '*G. aff. vittiformis*' but sister to G. alpestris. |
| *Galerina* | 55; *G. vittiformis* (Fr.) Singer | Monophyletic but no bootstrap support. Nested in clade with '*G. vittiformis*' and all of the '*G. aff. vittiformis*' but also *G. minima* and *G. alpestris*. Name applied because clade includes Smith type for *G. vittiformis f. bispora* but collections identified as *'vittiformis'* are not monophyletic. European and N. American. Spore number per basidium likely inconsistent (Gulden et al., 2005). |
| **Subgenus** *Tubariopsis* (Kühner ex Bas) A. H. Sm. & Singer emend. Gulden |  | Often on moss; distinctive microscopic characters, tibiiform cystidia, never pleurocystidia, many species lack clamps, spores often with minimal or no plage and are not dextrinoid (*G. stordalii* is a dextrinoid exception). Molecular phylogenies, here and from Gulden et al. (2005) support the monophyly of the subgenus in a sense close to that of Gulden & Hallgrímsson (2000). |
| *Tubariopsis* | 15; *G. arctica* (Singer) Nezdojm. | Supported clade of two Arctic samples. |
| *Tubariopsis* | 6; *G. dimorphocystis* A.H. Sm. & Singer  & *G. clavata* (Velen.) Kühner | Supported clade with two supported subclades. Subclade *G. dimorphocystis* (Smith and Singer, 1955) is the oldest name among names appearing in the first subclade: *G.* heterocystis/*G.* dimorphocystis/*G.* semilanceata. *G. semilanceata* described exclusively from PNW, but *G. dimorphocystis* and *G. heterocystis* are also described in PNW (and other locales, (Smith and Singer, 1964). Represented by 37 collections, almost all from western N. America. Subclade *G. clavata* represented by three specimens, all from Nordic Europe. Subclades may well be better viewed as separate species. |
| *Tubariopsis* | 63; *G. discreta* E. Horak, Senn-Irlet, Curti & Musumeci | One sequence |
| *Tubariopsis* | 39; *G. hybrida* Kühner | Supported clade of three, European and N. American |
| *Tubariopsis* | 61; *G. laevis* Singer | Supported clade of four sequences, European, consistently identified as *G.* laevis. |
| *Tubariopsis* | 62; *G. nigripes* A.H. Sm. & Singer | Sequence from specimen of *G. nigripes* examined by Smith and Singer (1964); clade also contains 2/21 specimens id'd as *G. heterocystis* - a similar species. |
| *Tubariopsis* | 24; *G. pseudocerina* sp. 1 | One sequence split by ABGD from supported clade of sequences identified as *G. pseudocerina*. All Nordic. |
| *Tubariopsis* | 25; *G. pseudocerina* sp. 2 | Two sequences split by ABGD from supported clade of sequences identified as *G. pseudocerina*. All Nordic. |
| *Tubariopsis* | 28; *G. stordalii* A.H. Sm. | Supported clade of 10 sequences, European |
| *Tubariopsis* | 47; *G. tibiicystis* (G.F. Atk.) Kühner | Supported, two sequences, European |
| *Tubariopsis* | 48; *G. tibiiformis* A.E. Wood | One sequence, Australian |
| **Subgenus** *Mycenopsis* A. H. Sm. & Singer emend. Berbee |  | *Mycenopsis* includes *Gymnopilus* with 88% support. Due to the small sample of *Gymnopilus* species included here, we do not propose name changes to recognize this relationship. *Mycenopsis* members usually grow on moss, thin, delicate mushrooms with conical or bell-shaped caps (Gulden et al., 2005), spores faintly ornamented or smooth and +/-calyptrate, cheilocystida only. Gulden et al. (2005) included Smith & Singer's Smith & Singer (1964) Sect. *Calyptrospora* in Sect *Mycenopsis*, as is supported in our phylogenies. Gulden & Hallgrímsson (2000) consider *Mycenopsis* to be a section in subgenus *Galerina*, but phylogenies here and in Gulden et al. (2005) suggest rather that *Mycenopsis* requires recognition as a subgenus because it is sister to a clade comprising *Naucoriopsis*, *Galerina*, and *Tubariopsis*. |
| *Mycenopsis* | 33; *G. allospora* A.H. Sm. & Singer | supported, two European sequences. |
| *Mycenopsis* | 35; *G. austrocalyptrata* A.H. Sm. & Singer | One sequence |
| *Mycenopsis* | Number not assigned; not grouped; short seq.; *G. calyptrospora* sp. | An unidentified species in Smith & Singer's (Smith and Singer, 1964) Sect. *Calyptrospora*. Originally identified as *G. subcerina* UNSW9931 collected in Australia. Due to geography and isolated phylogenetic position, likely a different species but close to *G. subcerina*. |
| *Mycenopsis* | 46; *G. calyptrospora* sp. | From Australia, identified as *G. subcerina* but does not match type of *G. subcerina* var. *subcerina*. |
| *Mycenopsis* | 52; *G. cephalotricha* Kühner | One sequence |
| *Mycenopsis* | 9; *G. fallax* A.H. Sm. & Singer | Supported; contains Gulden *G. fallax* sequences. Other names from UBC collections, but repeated DNA sequences were consistent; not suggestive of contamination. 9 sequences, Europe, N. America, Antarctica. |
| *Mycenopsis* | 38; *G. fibrillosa* A.H. Sm. | Smith holotype, one sequence |
| *Mycenopsis* | 17; *G. harrisonii* (Dennis) Bas & Vellinga | One Gulden sequence, near the *Gymnopilus* sequences |
| *Mycenopsis* | 40; *G. hypnorum* (Schrank) Kühner | Supported, four sequences all identified as '*G. hypnorum'*, N. America and Europe |
| *Mycenopsis* | 42; *G. lubrica* A.H. Sm. | Supported, contains Smith holotype, N. America and Europe |
| *Mycenopsis* | 19; *G. luteolosperma* A.H. Sm. & Singer  & *G. sphagnorum* (Pers.) Kühner | Supported clade with two supported subclades. Subclade with Gulden *G. luteolosperma* also includes a sequence from a UBC Ceska collection from Vancouver Island BC Canada. Subclade *G. sphagnorum*, 2 NO specimens. |
| *Mycenopsis* | 5; *G. mniophila* (Lasch) Kühner  & *G. pumila* var. *subalpina* A.H. Sm. | Supported clade with two supported subclades. Subclade with Gulden et al.'s (Gulden et al., 2005) *G. mniophila* also includes N. American, European samples. Subclade *G. pumila* var. *subalpina* includes Smith paratype sequence for the variety and only N. American sequences. However, *G. pumila* is an old European Persoon species (*Galerina pumila* (Pers.) Singer 1961) and collections from Europe identified as *G. pumila* come out in multiple other clades. Based on geography, variety *subalpina* is likely not monophyletic with the species' type. |
| *Mycenopsis* | Number not assigned; not grouped; short sequence; *G. mycenopsis* sp. | One sequence, N. American, identified as *G. fibrillosa* but does not match type. |
| *Mycenopsis* | 21; *G. paludosa* (Fr.) Kühner | Supported, four sequences all identified as '*G. paludosa'*, N. America and Europe |
| *Mycenopsis* | 26; *G. pumila* (Pers.) Singer | Single sequence, sister to group 4. Unclear which clade would correspond to the *G. pumila* type. As shown in our figure, sequences named *G. pumila* and *G*. *pumila* var. *subalpina* are not monophyletic. |
| *Mycenopsis* | 27; *G. sphagnicola* (G.F. Atk.) A.H. Sm. & Singer | Supported clade of two sequences, European, ID based on sequence from O73441 (Gulden et al., 2005) |
| *Mycenopsis* | 16; *G. subcerina* A.H. Sm. & Singer | Supported clade including Smith type sequence for *G. subcerina* var. *subcerina*. *G. subcerina* var. *subcerina's* description includes the type of *G. subcerina* so should be monophyletic with type. *G. subcerina* is an older name (Singer and Smith, 1958) than *G. calyptrata* (1960). Europe, N. America. |
| *Mycenopsis* | 4; *G. vexans* A.H. Sm. & Singer | Supported, sister to a Gulden *G. pumila* sequence. Named because it includes Smith paratype sequence. It also includes sequences identified as *G. pumila* (Gulden et al., 2005) but it is unclear which if any collections represent *G. pumila*, an old European name (*Agaricus pumilus* Pers. 1801). *Galerina vexans* could therefore be a synonym of *G. pumila*. |
| **Genus** *Gymnopilus*   P. Karst. | *Gymnopilus* | Like *Galerina*, ellipsoid or amygdaloid spores, roughened, with plage. Like many *Galerina* except *Tubariopsis*, *Gymnopilus* species have 'inflating dextrinoid/cyanophilic endospore' (Gulden et al., 2005). |
| *Gymnopilus* | 29; *Gymnopilus penetrans* (Fr.) Murrill | Two ITS sequences, only *Gy. penetrans* identified to species. |
| *Gymnopilus* | 30; *Gymnopilus punctifolius* (Peck) Singer | One sequence |
| *Gymnopilus* | 31; *Gymnopilus sp.* | One sequence |
| *Gymnopilus* | 32; *Gymnopilus spectabilis* (Weinm.) A.H. Sm. | One sequence |
| **Subgenus** *Sideroides* A. H. Sm. & Singer emend. Berbee |  | Often on rotten wood, spores blunt-ellipsoid, not or only faintly dextrinoid, usually smooth walled, with tibiiform cystidia (Smith and Singer, 1964). This well-supported infrageneric clade corresponds to a subset of similar species from stirps *Sideroides*, Smith & Singer (1964). Smith & Singer also included *G. pseudocamerina* and *G. larigna* in *Sideroides* but these species appear instead closer to *Naucoriopsis* in our phylogenies. Subgenus *Sideroides* is represented by two sequences from Europe and many collections from British Columbia, Canada. In our consensus and RPB2 phylogenies, *Sideroides* is sister to *Psilocybe*, but with little bootstrap support. *Sideroides* plus *Psilocybe* is sister to a clade consisting of the other four subgenera of *Galerina*, thus justifying elevation to subgenus and perhaps, with future support by additional data, recognition at higher taxonomic rank. |
| *Sideroides* | 53; *G.* aff. *sideroides* | Supported, 12 sequences, all UBC specimens from BC Canada, 9/12 samples identified as *G. sideroides* (3 as *G. stylifera*). *G. sideroides* is an old, European, name (*Agaricus sideroides* Bull. 1793) and may not be applicable to this clade that at present is known only from N. America. |
| *Sideroides* | 7; *G*. aff. *stylifera* | Supported sister clade to *G. stylifera*. North American. |
| *Sideroides* | 14; *G. mammillata* (Murrill) A.H. Sm. & Singer | Supported, 11 sequences, all from all UBC Ceska specimens from western BC Canada. Smith describes *G. mammillata* as drying to white (vs. *G. sideroides* drying to brown); most of the dry UBC samples are whitish. |
| *Sideroides* | 60; *G. pseudobadipes* Joss. | Supported sister clade to *G. mammillata*; two European specimens identified as *G. pseudobadipes* O154252 and *G. stylifera* x6920 |
| *Sideroides* | 8; *G. stylifera* (G.F. Atk.) A.H. Sm. & Singer | Supported, 11 sequences. *G. stylifera* is an American species (New York, G.F. Atk. 1918). Smith type sequences for *G. stylifera* var. *caespitosa* A.H. Sm. & Singer(Smith and Singer, 1964) and *G.* stylifera var. *badia* A.H. Sm. & Singer  (Singer and Smith, 1958) are part of this monophyletic group. One collection from France, others from N. America. |
| **Genus** *Psilocybe* (Fr.) P. Kumm. | 11; *Psilocybe caerulipes* (Peck) Sacc. | One sequence |
| *Psilocybe* | 12; *Psilocybe cubensis* (Earle) Singer | One sequence |
| *Psilocybe* | 13; *Psilocybe cyanescens* Wakef. | One sequence |

References

**Gulden, G., & Hallgrímsson, H.** 2000. The genera *Galerina* Earle and *Phaeogalera* Kühner (Basidiomycetes, Agaricales) in Iceland. *Acta Botanica Islandica* 13:3-54.

**Gulden, G., Stensrud, O., Shalchian-Tabrizi, K., & Kauserud, H.** 2005. *Galerina* Earle: A polyphyletic genus in the consortium of dark-spored agarics. *Mycologia* 97:823-837.

**Singer, R., & Smith, A.H.** 1958. Mycological investigations on Teonanácatl, the Mexican hallucinogenic mushroom. Part II. A taxonomic monograph of *Psilocybe*, section *Caerulescentes*. *Mycologia* 50:262-303.

**Smith, A.** 1953. New species of *Galerina* from North America. *Mycologia* 45:892-925.

**Smith, A., & Singer, R.** 1955. New species of *Galerina*. *Mycologia* 47:557-596.

**Smith, A.H., & Singer, R.** 1964. A Monograph on the Genus *Galerina* Earle. Hafner, New York.
