# Supplementary material for: Phylogenetic analysis of the distribution of deadly amatoxins among the little brown mushrooms of the genus Galerina
Source: PLoS One. 2021 Feb 10;16(2):e0246575. doi: 10.1371/journal.pone.0246575 (PMC7875387; doi:10.1371/journal.pone.0246575)
Supplement: Attachment — Submitted filename: Landry.rebuttal.docx [file pone.0246575.s009.docx]

On the following pages, we provide details about our response to reviewer comments in green. We use blue font to quote revisions to our text.

**Comments to the Author**

1. Is the manuscript technically sound, and do the data support the conclusions?

The manuscript must describe a technically sound piece of scientific research with data that supports the conclusions. Experiments must have been conducted rigorously, with appropriate controls, replication, and sample sizes. The conclusions must be drawn appropriately based on the data presented.

Reviewer #1: Yes

Reviewer #2: Yes

2. Has the statistical analysis been performed appropriately and rigorously?

Reviewer #1: Yes

Reviewer #2: Yes

3. Have the authors made all data underlying the findings in their manuscript fully available?

The [PLOS Data policy](http://www.plosone.org/static/policies.action#sharing) requires authors to make all data underlying the findings described in their manuscript fully available without restriction, with rare exception (please refer to the Data Availability Statement in the manuscript PDF file). The data should be provided as part of the manuscript or its supporting information, or deposited to a public repository. For example, in addition to summary statistics, the data points behind means, medians and variance measures should be available. If there are restrictions on publicly sharing data—e.g. participant privacy or use of data from a third party—those must be specified.

Reviewer #1: Yes

Reviewer #2: Yes

4. Is the manuscript presented in an intelligible fashion and written in standard English?

PLOS ONE does not copyedit accepted manuscripts, so the language in submitted articles must be clear, correct, and unambiguous. Any typographical or grammatical errors should be corrected at revision, so please note any specific errors here.

Reviewer #1: Yes

Reviewer #2: Yes

5. Review Comments to the Author

Please use the space provided to explain your answers to the questions above. You may also include additional comments for the author, including concerns about dual publication, research ethics, or publication ethics. (Please upload your review as an attachment if it exceeds 20,000 characters)

Reviewer #1: The manuscript describes a phylogenetic and toxicologic analysis of mushrooms in the genus Galerina, which contains deadly poisonous species that produce amatoxins. It is the most complete analysis of Galerina phylogeny in the context of toxicity to date, complementing but considerably expanding previous work by other authors. The analyses are appropriate and follow standard practices in fungal molecular phylogeny and in amatoxin analysis from mushroom tissue; the corresponding author is well-versed in fungal phylogeny, while the first author was trained in amatoxin analysis in the premier lab for this technique. The paper contributes to our knowledge and is well worth publishing. A few minor edits and suggestions are given below.

Line 62: Suggest “Following treatment , he slowly recovered” (“After that” sounds more spontaneous than was actually the case)

Done.

Line 64: as common names are variable, suggest adding “(Armillaria species)” after “honey mushrooms”

Done.

Lines 179-181: suggest rephrasing as “…Galerina (Strophariaceae), other Strophariaceae, and members of closely related families Hymenogastraceae, Crepidotaceae…” or some other phrasing that indicates which family Galerina is in.

Done. Revised as:

*Galerina*, *Hebeloma* and *Gymnopilus*, genera representing the family Hymenogastraceae.

Line 247: “broader or narrower”

Done.

Line 280: “amatoxin-positive”

Done
Line 660: “Hallen-Adams HE”, not “Len-Adams HEH”

Corrected

Figures S1 and S2: The bright aqua text used to indicate the types is effectively illegible (as displayed on my computer). Suggest a slightly darker color.

Agreed. We now use a darker blue that shows up better.

Reviewer #2: The manuscript by Brandon Landry and colleagues uses chemical analyses (HPLC/MS), DNA barcoding, and molecular phylogenetic analyses to determine the phylogenetic placement of species in the genus Galerina that produce deadly amatoxins. The manuscript is well written overall, its methods are suitable to its objectives, and interpretation of data is logical. I am including some specific recommendations below that I would ask the authors to address, but I do not find that any major revisions are necessary.

The largest suggestion that I would make of a more general nature is to make a more active contribution to clarifying the infrageneric taxonomy of the genus. The authors stated that this is the most large-scale phylogeny of the genus to–date. This therefore seems like an excellent opportunity to clarify infrageneric nomenclature. However, even within the manuscript, these concepts seem a bit fuzzy. For example, lines 86-88 refer to "Naucoriopsis", "Galerina", "Tubariopsis", and "Mycenopsis" as provisional clade names, but lines 328-330 and line 522, for example, refer to sections, suggesting that these names have a formal taxonomic designation. At the very least, the manuscript should be consistent about referring to specific subgeneric taxa either using provisional clade names or formal sections, but not switching back and forth. Even better would be to assist in formalizing the taxonomy by establishing validly published sectional names where none exist and more clearly confirming or refuting existing formal names rather than perpetuating nomenclatural confusion where clarity could be added.

Done. Table S3 now shows that we are formally emending infrageneric taxa that have been recognized at various ranks to subgenera.

This was a good suggestion because it simplifies the story while imposing minimal nomenclatoral change. We base most of the subgenera on clades already recognized by Gulden & Hallgrímsson 2000. The subgenera are consistent with informal clade names in the phylogeny of the LSU region from Gulden et al. 2005.


Line 19 uses the term “amatoxins,” whereas line 25 uses the term “amanitins”; I recommend being consistent with terms or at least briefly describe the difference in the beginning so the reader knows why a particular term is being used at any given point.

Replaced 'amanitins' with 'amatoxin' when referring to general group of toxins, applying 'amanitin' when referring to a specific compound, e.g. β-amanitin.

Added explanation: Prompted by these poisoning cases, Tyler and Smith [10] used paper chromatography to show that *G. venenata* contains α- and β-amanitin *– two of the amatoxins,* the toxic peptides identified from the genus *Amanita*.

Line 95: should citation 19 be included here as well?

Not done because ref 19 is no longer appropriate due to edits that changed context.

Line 115: recommend replacing "narrower species that are uncorrelated with morphological characters" with "cryptic species".

Done. Is now shorter and more direct.

line 121: recommend changing "without Bayesian support" to "without strong Bayesian support" (there must be some support, or the clades would not be resolved).

Done.

This is also an issue in a number of other places in the manuscript, including lines 344, 381, 411 and perhaps elsewhere. Specify what level you are referring to, such as “support lower than 50%”, as “no support” only makes sense if the bootstrap support value is 0%.

Done as specified in italics below.

Line 267: proportion of putative species that formed clades with *moderate to high bootstrap support* of *70% or more*,
Line 346: monophyletic, many with *>70%* bootstrap support

Line 353: Internal bootstrap support values *>70%*

Line 391: two clades receiving *>90%* bootstrap support

Line 414: *Naucoriopsis* and *Galerina*, although with *<50%* bootstrap support

Line 482: although with bootstrap support *<50%*

Line 167: recommend giving this primer a name so that others using it in the future can refer to it by name rather than "the redesigned internal forward primer reported by Landry et al."

It is now berniF, named for Berni van der Meer who designed it.

line 181, 282, and elsewhere (many places throughout manuscript): recommend replacing "S1 table" with "table S1”, “S2 table” with ‘’table S2” etc.

Not changed because "S1 Table" is the journal-specified format.

line 182: recommend replacing “online server with the L-INS setting server” with “online server with the L-INS setting”

ln 183, Done

line 185: recommend replacing “best models” with “best nucleotide substitution models”

Done. we selected as best nucleotide substitution models

Lines 186-187 and line 200: please clarify whether RPB2 sequences were trimmed at the 3’ end. If sequences include the region between the binding sites of reverse primers 7R and 7.1R, there is an intron that should be accounted for in the jModelTest and RAxML partitions.

I double-checked and the intron had been spliced out of our alignment before analysis.

This means that the text was OK as originally submitted:

Line 184: For the *RPB2* dataset, we excluded introns from the final alignment.

Line 190: recommend replacing “patterns of support over the topologies” with simply “branch support”.

Line 191 Done. We used 500 bootstrap replicates to assess branch support.

Lines 236-237: Please include a description of the basis for assignment of the beta-amanitin peak, given that no standard was included.

We add an explanation that the basis is retention time, under the conditions used for chromatography of mushroom amanitins, and we added a citation of the paper that best documents the positions of the amanitin peaks under the conditions used in our paper.

Samples were recorded as positive for β-amanitin based on a peak with the retention time of 8.0 minutes that is expected under the chromatography conditions used [31].

Line 246: recommend replacing “multiple hits” with “multiple substitutions”

Line 269 done.

Line 280: recommend replacing “amatoxins-positive” with “amatoxin-positive”
Line 307 done.

Lines 314-315: “from genera closely allied with Galerina, from the four from Gymnopilus sp” reads awkwardly; suggest rewording.

Line 316. Reworded:

Amatoxins were not found in any of the genera closely related to *Galerina;* amatoxins were not detected from the four *Gymnopilus* spp., the three samples of *Hebeloma* or the sample of *Flammula alnicola*. (Figs 1 and 2).

Line 316: expand G. badipes to Galerina badipes, since Gymnopilus was mentioned in the previous line.
Done.

Line 344: recommend replacing “S1-S3 Figs” with “Figs. S1-S3”
Not changed because "S1 Figs" is the journal-specified format.

Lines 344-345 and line 385: “application of names is confused and inconsistent”. The word “confused” seems unnecessarily pejorative to me; consider “inconsistent” perhaps as a substitute? It would be more informative anyway to describe the problem more specifically — are these taxonomic issues (taxa were poorly delimited in the first place), or issues of application (e.g., misidentifications because the taxa are difficult to distinguish morphologically or because the specimens were incompletely examined)?

Corrected and we agree that pejorative implications are unwarranted. We don't discuss the reasons for the lack of congruence because we don't have detailed morphological notes for most specimens that would allow us to go further.

Line 381 ...application of names to species-level clades is inconsistent (S1 Fig). The inconsistency of species-level identifications even by specialists in the genus points to lack of congruence between morphological characters and genetically defined species.

Line 359: include a comma between “Grant's Pass” and “Oregon”
Done

Line 393: no comma necessary after “G. vittiformis”
Done

Lines 523-524: recommend replacing “new RPB2 remove” with “new RPB2 sequences remove”
Done
Line 530: recommend replacing “nesting” with “nested”
Done

Line 577: G. marginata s.l. does not appear to be designated with a black line in Fig. S2 like the caption suggests.

Line 604: Two-letter country codes should be provided with the table.
S1 Table does specify the two-letter country codes in column D.
Table 1: Sideroides should be capitalized.

Done
Fig. 1. Specify that “spp.” designates genera whereas names without “spp.” designate infrageneric groups in Galerina.

Thanks, corrected.

Clade colors correspond to *Galerina* subgenera or to species of *Gymnopilus* and *Psilocybe* that appear nested in *Galerina* (S3 Table).

Fig. 4. Image quality is fairly low. Consider using image stacking to obtain a better image.
The images do clearly show the features that are important in recognizing toxic species, and so we would have difficulty improving them at this point due to covid-related problems with access to the herbarium/specimens and to the camera.

Fig S1: Based on the dark black line, G. patagonica, G. physospora, and G. sulciceps should be treated in G. marginata s.l., but the Discussion (lines 436-442) suggests that this is not the case; this point should be made consistent one way or the other.

We do consider all of those species to be part of *G. marginata* s.l. and clarify this as follows:

line 440 Also in *G. marginata* s.l., in *Naucoriopsis*, and also toxin-positive, *Galerina sulciceps...*

line 445 *G. patagonica*, also in the *G. marginata* *s.l.* species complex

6. PLOS authors have the option to publish the peer review history of their article ([what does this mean?](https://journals.plos.org/plosone/s/editorial-and-peer-review-process#loc-peer-review-history)). If published, this will include your full peer review and any attached files.


If you choose “no”, your identity will remain anonymous but your review may still be made public.


**Do you want your identity to be public for this peer review?** For information about this choice, including consent withdrawal, please see our [Privacy Policy](https://www.plos.org/privacy-policy).

Reviewer #1: **Yes:** Heather E Hallen-Adams

Reviewer #2: **Yes:** Todd Osmundson

[NOTE: If reviewer comments were submitted as an attachment file, they will be attached to this email and accessible via the submission site. Please log into your account, locate the manuscript record, and check for the action link "View Attachments". If this link does not appear, there are no attachment files.]

While revising your submission, please upload your figure files to the Preflight Analysis and Conversion Engine (PACE) digital diagnostic tool, <https://pacev2.apexcovantage.com/>. PACE helps ensure that figures meet PLOS requirements. To use PACE, you must first register as a user. Registration is free. Then, login and navigate to the UPLOAD tab, where you will find detailed instructions on how to use the tool. If you encounter any issues or have any questions when using PACE, please email PLOS at [figures@plos.org](mailto:figures@plos.org). Please note that Supporting Information files do not need this step.
