# Supplementary material for: Phylogenetic analysis of the distribution of deadly amatoxins among the little brown mushrooms of the genus Galerina
Source: PLoS One. 2021 Feb 10;16(2):e0246575. doi: 10.1371/journal.pone.0246575 (PMC7875387; doi:10.1371/journal.pone.0246575)
Supplement: Attachment — Submitted filename: Landry.rebuttal.2021.Jan20.docx [file pone.0246575.s010.docx]

The reviewers pointed out typos or places where clarification was needed. None were complicated and all were helpful. We made all of the corrections as recommended.

Reviewer #1: A tiny point: Line 98: there appears to be an extra “G.” in “G. G. beinrothii”.

Done.

I will now include this sentence because it tells me I have not met the character count.

Reviewer #2: I commend Brandon Landry and colleagues on their careful revision of the manuscript. The authors addressed all comments with either corrections or reasonable justifications. The nomenclatural details that I mentioned in the previous review are handled very well in the revision. I include a few minor points below that I think could improve clarity or fix typographic errors. The manuscript provides a great contribution to our understanding of amatoxin evolution in Galerina, and improves the predictive framework for determining which other species in the genus are toxic. I look forward to seeing how these predictions perform when toxicity data are collected for additional species. In addition, the paper improves the resolution of infrageneric relationships in Galerina, and points to some areas where further phylogenetic studies are needed (such as the relationship between Galerina subgen. Mycenopsis and Gymnopilus, and the relationship between Galerina subgen. Sideroides and Psilocybe) -- it will be interesting to see how these questions are resolved with future research.

Line 44: italicize Galerina

Done.

Line 57: recommend replacing “on liver” with “on the liver”

Done.

Line 84: suggest “subgenera and sections; e.g., Gulden and Hallgrímsson [11], and Smith and Singer [12].”

Done.

Line 98: recommend replacing “G. G. beinrothii” with “G. beinrothii”

Done.

Line 161: missing period after “amplifications”

Done.

Line 424: recommend replacing “without support” to “without strong support”

Done.

Line 426: recommend replacing “relations” with “relationships”

Done.

Line 442: G. sulciceps is reported here as toxin-positive, but that result is not shown in Fig. 2 or Fig. S1 or Table S1; I assume that the information on toxicity is therefore from the literature and that the sequenced specimen is not the one that was tested. I recommend clarifying this point, along with a brief explanation of the basis for considering the reported specimen and the sequenced specimen as conspecific.

Done.

Also in *G. marginata* s.l., in *Naucoriopsis*, and reported as toxin-positive [36], *Galerina sulciceps* is a tropical species found in greenhouses. Toxin tests and DNA sequence barcodes are not yet available for the same collection of *G. sulciceps.*

Line 449: Similarly, I recommend clarifying this line, replacing “Other toxin-positive specimens without DNA barcodes” to something like “Other species reported in the literature as toxin-positive, but not included in our molecular analyses due to lack of DNA sequence data”

Done, edited as:

Three species reported in the literature as toxin-positive, *Galerina beinrothii* [16], *G. helvoliceps* and *G. fasciculata* [14, 15], could not be included in our molecular analyses due to lack of DNA sequence data.

Line 452: consider appending something like “These results further support our conclusion that the amatoxin-producing Galerina species are found within the G. marginata s.l. species complex in subgenus Naucoriopsis.”

Done.

Line 453: recommend replacing “from the G. marginata s. l.” with “from G. marginata s. l.”

Done.
